# Thermal Properties of Zeolite-Containing Composites

**DOI:** 10.3390/ma11030420

**Published:** 2018-03-13

**Authors:** Taro Shimonosono, Yoshihiro Hirata, Kyohei Nishikawa, Soichiro Sameshima, Kenichi Sodeyama, Takuro Masunaga, Yukio Yoshimura

**Affiliations:** 1 Department of Chemistry, Biotechnology, and Chemical Engineering, Kagoshima University, Kagoshima 890-0065, Japan; shimonosono@cen.kagoshima-u.ac.jp (T.S.); k1599051@kadai.jp (K.N.); samesima@cen.kagoshima-u.ac.jp (S.S.); 2 Shirasu R&D Laboratory, Regional Resource Division, Kagoshima Prefectural Institute of Industrial Technology, Kagoshima 899-5105, Japan; sodeyama@kagoshima-it.go.jp (K.S.); masunaga@kagoshima-it.go.jp (T.M.); yosiyuki@kagoshima-it.go.jp (Y.Y.)

**Keywords:** thermal conductivity, zeolite, phenol resin, shirasu glass, composite

## Abstract

A zeolite (mordenite)–pore–phenol resin composite and a zeolite–pore–shirasu glass composite were fabricated by hot-pressing. Their thermal conductivities were measured by a laser flash method to determine the thermal conductivity of the monolithic zeolite with the proposed mixing rule. The analysis using composites is useful for a zeolite powder with no sinterability to clarify its thermal properties. At a low porosity <20%, the thermal conductivity of the composite was in excellent agreement with the calculated value for the structure with phenol resin or shirasu glass continuous phase. At a higher porosity above 40%, the measured value approached the calculated value for the structure with pore continuous phase. The thermal conductivity of the monolithic mordenite was evaluated to be 3.63 W/mK and 1.70–2.07 W/mK at room temperature for the zeolite–pore–phenol resin composite and the zeolite–pore–shirasu glass composite, respectively. The analyzed thermal conductivities of monolithic mordenite showed a minimum value of 1.23 W/mK at 400 °C and increased to 2.51 W/mK at 800 °C.

## 1. Introduction

Zeolite has a framework of aluminosilicate with defined sizes of channels and cavities in the range of 0.2–1.0 nm, and has been used in wide applications such as molecular sieves, catalysts, heat pumps, and hydrogen storages. To control the transport of energy in the system with zeolite, the thermal conductivity of zeolite is a key factor. Since zeolite powders have little sinterability, the measurement of the thermal conductivity of dense bulk zeolite is very difficult. Coquil et al. synthesized pure silica zeolite (PSZ) films by spin-coating a zeolite suspension onto silicon substrates, followed by calcination and vapor-phase silylation [1]. The cross-plane thermal conductivity of the PSZ films of 40% porosity is reported to be 1.02 ± 0.10 W/mK at room temperature [1]. In molecular dynamics simulations, McGaughey et al. predicted the thermal conductivity of 3.53 W/mK for sodalite, 2.07 W/mK for faujasite, and 1.68 W/mK for zeolite-A at 27 °C [2]. Griesinger et al. measured the thermal conductivity of a zeolite-NaA powder compact and compared it with a mixing rule which treats the summation of heat transported through the solid phase, gas phase, and those mixed phases, resulting in 3.3 W/mK for the thermal conductivity of zeolite-NaA at 150 °C [3]. The above research results suggest the possible thermal conductivities of 1–4 W/mK for bulk zeolite.

In this paper, the thermal conductivities of the zeolite–phenol resin composite and the zeolite–shirasu glass composite were measured to evaluate the thermal conductivity of zeolite with a mixing rule proposed by Hirata [4]. A kind of volcanic ejecta, shirasu, consists of volcanic glass (about 70 mass %) and crystalline phases (about 30 mass %) such as plagioclase, quartz, and pyroxene [5]. In this paper, the volcanic glass (shirasu glass) was used to make the composites with zeolite. The thermal conductivity for the structure consisting of a continuous phase 2 and a dispersed phase 1 is expressed by Equation (1) [4]:

(1) $$\kappa_{\mathrm{ap}}{=\kappa }_{2}-\kappa_{2}V_{a}^{2/3}\left[1-\frac{1}{1-V_{a}^{1/3}(1-{(\kappa }_{2}{/\kappa }_{1}))}\right]$$

where *κ*_1_ and *κ*_2_ are the thermal conductivities of dispersed and continuous phases, respectively, and *V*_a_ is the volume fraction of a dispersed phase 1 in the two phase system (*V*_a_ = *V*_1_/(*V*_1_ + *V*_2_)). This mixing rule can be expanded to the three phase system by treating the thermal conductivity of the two phase system as a new continuous phase, as expressed by Equation (2):

(2) $$\kappa_{b}{=\kappa }_{\mathrm{ap}}-\kappa_{\mathrm{ap}}V_{3}^{2/3}\left[1-\frac{1}{1-V_{3}^{1/3}(1-{(\kappa }_{\mathrm{ap}}{/\kappa }_{3}))}\right]$$

where *κ*_3_ and *V*_3_ are the thermal conductivity and volume fraction of a third phase dispersed. That is, no limitation of the number of phases in the solid material is included in the proposed mixing rule. Fortunately, the zeolite particles have no sinterability and can be treated as the dispersed phase 3 in the mixing rule even if the zeolite fraction is high. The phenol resin and shirasu glass soften by heating and form the continuous phase containing the dispersed zeolite particles. The pores in the composite can be also treated as the dispersed phase 1 in the porosity range of 0–25 vol % [6,7]. The thermal conductivities of the zeolite-containing glass composites in this paper were measured at 25–800 °C and analyzed by the mixing rule by Equations (1) and (2).

## 2. Experimental Procedure 

The mordenite-type zeolite was used to prepare dense composites (HSZ-660HOA, Tosoh Co. Ltd., Tokyo, Japan). The specific surface area after drying at 100 °C and true density after drying at 600 °C for the mordenite were 121.6 ± 0.4 m^2^/g and 2.192 ± 0.01 g/cm^3^, respectively. The chemical composition of the zeolite was analyzed at Kagoshima Prefectural Institute of Industrial Technology in Kagoshima, Japan, and was 86.04 mass % SiO_2_, 5.34 mass % Al_2_O_3_, 0.35 mass % Na_2_O, 0.09 mass % K_2_O, 0.03 mass % MgO, 0.02 mass % TiO_2_, and 8.12 mass % Ig. loss at 1050 °C. The SiO_2_/Al_2_O_3_ molar ratio was calculated to be 27. The phenol resin (PhenoCure) was supplied by Buehler Co., Lake Bluff, IL, USA, and the true density after drying at 100 °C was 1.324 ± 0.02 g/cm^3^. The shirasu glass powder was supplied by Kagoshima Prefectural Institute of Industrial Technology in Japan (SRG5J3, chemical composition (mass %): SiO_2_ 73.7, Al_2_O_3_ 12.2, K_2_O 4.38, Na_2_O 3.64, Fe_2_O_3_ 1.57, CaO 1.44, MgO 0.28, TiO_2_ 0.21, MnO 0.05, P_2_O_5_ 0.04, Ig. loss 2.49). The specific surface area after drying at 100 °C and true density after drying at 600 °C for the shirasu glass were 2.52 ± 0.01 m^2^/g and 2.330 ± 0.01 g/cm^3^, respectively. In the fabrication of the zeolite–pore–phenol resin composite, the zeolite was mixed with phenol resin at the volume fractions of 0–28.2 vol % and ball-milled for 24 h with α-Al_2_O_3_ balls, 3 mm in diameter. The mixed powder was hot-pressed at 150 °C under 10 MPa in air for 20 min (SimpliMet 2, Buehler Co., Lake Bluff, IL, USA), and the disk-shaped composites, 25.4 mm in diameter and 10 mm thick, were fabricated. In the fabrication of the zeolite–pore–shirasu glass composites, the mixed powders at the zeolite fractions of 0–41.1 vol % were dispersed in double-distilled water at the solid content of 30 vol %. The pH value of the suspension was adjusted to 8–9, and the suspension was consolidated on a gypsum board to a disk shape, 10 mm in diameter. The consolidated compact was hot-pressed at 800–950 °C under 39 MPa in an Ar atmosphere for 2 h (FVPHP-5-R, Fuji Dempa Kogyo Co. Ltd., Osaka, Japan). The fabricated composites were polished with a SiC abrasive paper and were annealed at 600 °C in air for 10 h. The density of the fabricated composite was measured by the Archimedes method using double-distilled water. The phases produced in the composites were identified by X-ray diffractometer (RINT2200, Rigaku Co. Ltd., Tokyo, Japan). The microstructure was observed using a scanning electron microscope equipped with an energy dispersive X-ray spectrometer (SEM-EDX, Quanta400, Thermo Fisher Scientific Inc., Waltham, MA, USA). The thermal properties (heat capacity, thermal diffusibility, and thermal conductivity) were measured by a laser flash method at Okayama Ceramics Research Foundation in Japan (1406-18 Nishikatakami, Bizen-shi, Okayama, Japan).

## 3. Results and Discussion

### 3.1. Mordenite–Pore–Phenol Resin Composites

**Figure 1:**
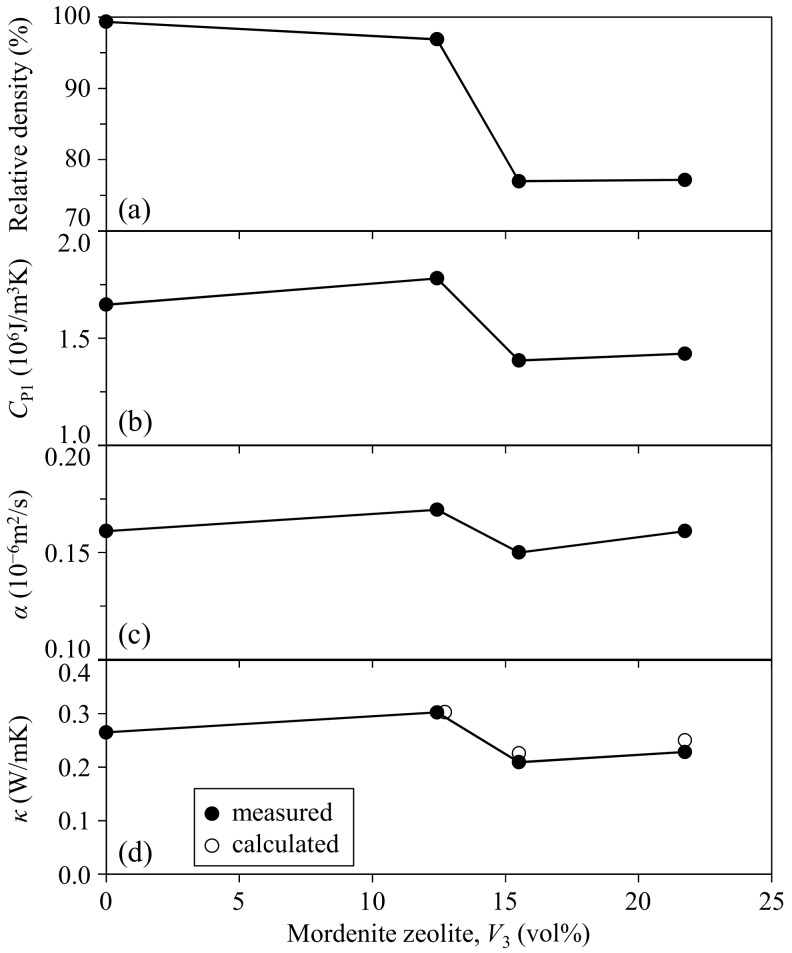
(**a**) Relative density; (**b**) heat capacity; (**c**) thermal diffusibility; and (**d**) thermal conductivity of the mordenite zeolite–pore–phenol resin composites at room temperature. The open circles show the thermal conductivity calculated by Equation (2) in text with 3.63 W/mK for mordenite zeolite.

Only the mordenite phase was identified in the composite by X-ray diffractometer after hot-pressing at 150 °C. Figure 1 shows the relative density, heat capacity, thermal diffusibility, and thermal conductivity of the zeolite–pore–phenol resin composites as a function of zeolite fraction. The relative density of the composite was 96.9–99.3% at the zeolite volume fractions of 0–12.4 vol %, and decreased to 77.0–77.2% at the zeolite fractions of 15.5–21.7 vol %. The heat capacity of the composite well reflected the relative density, and this result suggests that the pores included in the composite had a large effect on the heat capacity (*C*_P1_). However, the influence of porosity on the thermal diffusibility (*α*) and thermal conductivity (*κ*, product of *C*_P1_ and *α* values) was relatively small. The measured thermal conductivities of the zeolite–pore–phenol resin composites were compared with the values calculated by Equation (2). The thermal conductivities of monolithic phenol resin and zeolite particles were determined as follows. The thermal conductivity of 100% phenol resin was determined to be 0.271 W/mK by Equation (1) using the measured thermal conductivities of the dense phenol resin (0.3% porosity, 0.265 W/mK) and air (0.0265 W/mK at 25 °C [8]). The thermal conductivity of the mordenite zeolite was calculated to be 3.63 W/mK by Equation (2) using the measured *κ* value (0.303 W/mK) for the 12.4 vol % zeolite–3.1 vol % pore–phenol resin composite (mordenite: dispersed phase 3 in Equation (2)). The values of 0.271 W/mK for phenol resin, 0.0265 W/mK for air, and 3.63 W/mK for mordenite zeolite were used for the calculation of *κ* values for the 15.5–21.7 vol % zeolite–22.8–23.0 vol % pore–phenol resin composites. The measured *κ* values in Figure 1d (0.209 and 0.229 W/mK for 15.5 and 21.7 vol % zeolite, respectively) were well explained by the calculated *κ* values (0.226 and 0.250 W/mK for 15.5 and 21.7 vol % zeolite, respectively).

The small effect of porosity on the measured *κ* values at 16–22 vol % zeolite in Figure 1d is explained by the compensation effect between the mordenite zeolite with a higher *κ* value (3.63 W/mK) and air (in pores) with a lower κ value (0.0265 W/mK). The calculated thermal conductivity of the mordenite zeolite in this paper was comparable to those calculated by molecular dynamics simulation (3.53 W/mK for sodalite, 2.07 W/mK for faujasite, and 1.68 W/mK for zeolite-A at 27 °C [2]), as well as close to the calculated thermal conductivity of 3.3 W/mK for zeolite-NaA at 150 °C [3]. The result in this paper indicates the useful approach based on the comparison between measured and calculated thermal conductivities of a zeolite-containing composite to evaluate the thermal conductivity of monolithic zeolite.

### 3.2. Microstructure of the Mordenite–Pore–Shirasu Glass Composites

**Figure 2:**
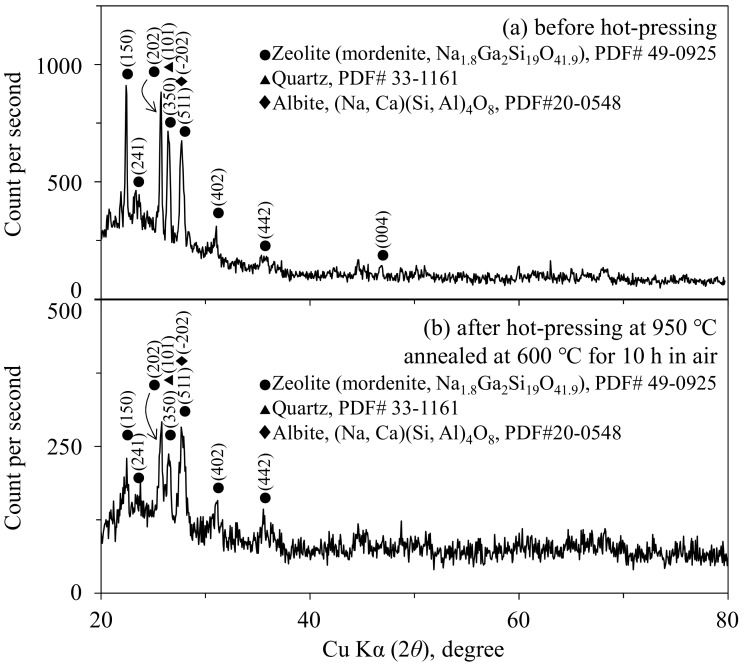
Phases of the 18.2 vol % zeolite–12.4 vol % pore–shirasu glass composite (**a**) before and (**b**) after hot-pressing at 950 °C for 2 h in an Ar atmosphere.

Figure 2 shows the X-ray diffraction patterns of the 18.2 vol % mordenite zeolite–12.4 vol % pore–shirasu glass composite before and after hot-pressing at 950 °C. The quartz and albite phases before hot-pressing were included in the shirasu glass used. As seen in Figure 2b, no change of the crystalline phases was recognized after hot-pressing at 950 °C, indicating little chemical interaction between mordenite and shirasu glass.

**Figure 3:**
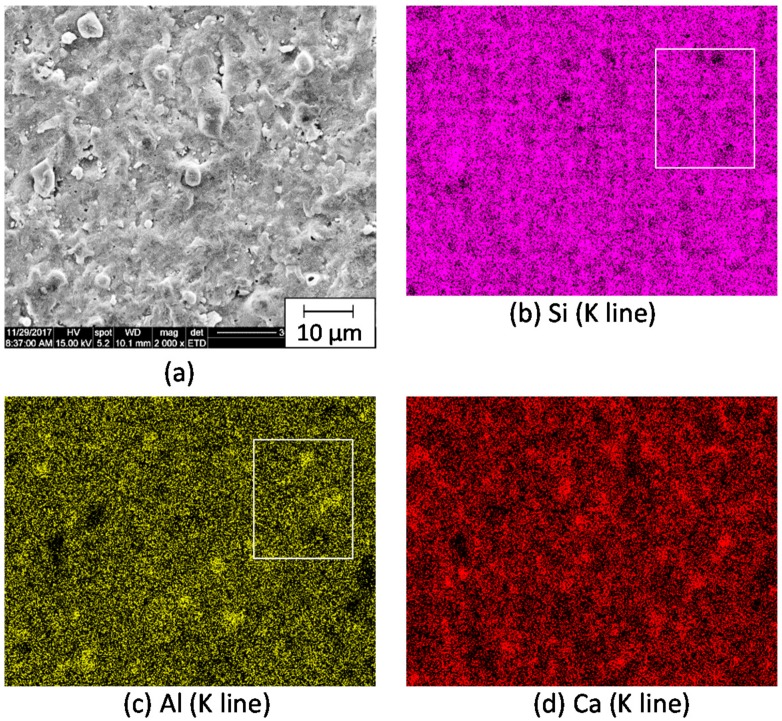
(**a**) Microstructure and (**b**–**d**) elementary distributions of the shirasu glass with 1.1% porosity, hot-pressed at 950 °C and annealed at 600 °C.

Figure 3 shows (a) the microstructure and (b–d) elementary distributions of the shirasu glass with 1.1% porosity, hot-pressed at 950 °C and annealed at 600 °C. The overlap of concentrated distributions of Al and Ca elements may reflect the albite phase, (Na, Ca)(Si, Al)_4_O_8_, detected in the shirasu glass by X-ray diffraction. The chemical composition (mass %) for the whole area of Figure 3a was examined by EDX (energy dispersive X-ray spectrometry) as follows: 1.93 Na, 2.18 K, 13.91 Ca, 5.62 Al, 36.48 Si, 22.29 O, 17.60 C, 100.01 Total. The measured mass ratio of Al/Si was 0.154 and close to the Al/Si ratio (0.187) on the basis of chemical analysis of the shirasu glass powder. Similarly, the Al/Si ratio for the square part in Figure 3b,c was 0.142. The C element detected may come from the carbon plates used or CO gas produced in the hot-pressing apparatus and will be studied further.

**Figure 4:**
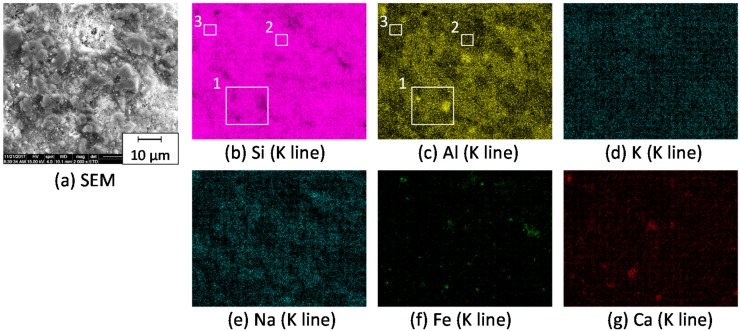
(**a**) Microstructure and (**b**–**g**) elementary distributions of the composite with 35.3 vol % zeolite–14.2 vol % porosity, hot-pressed at 950 °C and annealed at 600 °C.

Figure 4 shows the microstructure and elementary distribution of the composite with 35.3 vol % zeolite and 14.2 vol % porosity, hot-pressed at 950 °C and annealed at 600 °C. The microstructure is characterized by two different assemblies of grains. One is the larger grains-connected area, seen in square 1 and square 3, and the other is the smaller grains-agglomerated area (square 2 in Figure 4b). In the square 1 and square 2 structures, high and low concentrations of Al element were measured, respectively. In addition, the Al distribution showed good agreement with the distribution of Na element. The mass ratio of Al/Si in square 1 was 0.153, and close to 0.187 for the shirasu glass powder. The overlap of the distributions of Na and Al elements and the Al/Si mass ratio in square 1 indicate the shirasu glass continuous phase with albite phase. On the other hand, the mass ratio of Al/Si in square 2 was 0.098, which was close to the Al/Si mass ratio of the zeolite powder (0.0703). Similarly, the chemical composition of another part surrounded by square 3 in Figure 4b was examined by EDX as follows: 1.37 Na, 2.51 K, 0.31 Ca, 0.64 Fe, 4.18 Al, 38.68 Si, 44.42 O, 7.94 C, 100 Total. The measured mass ratio of Al/Si was 0.106, which was close to the ratio of Al/Si of the zeolite (0.0703). The C element was also detected in the zeolite–shirasu glass composite.

### 3.3. Thermal Properties of the Mordenite–Shirasu Glass Composites

**Figure 5:**
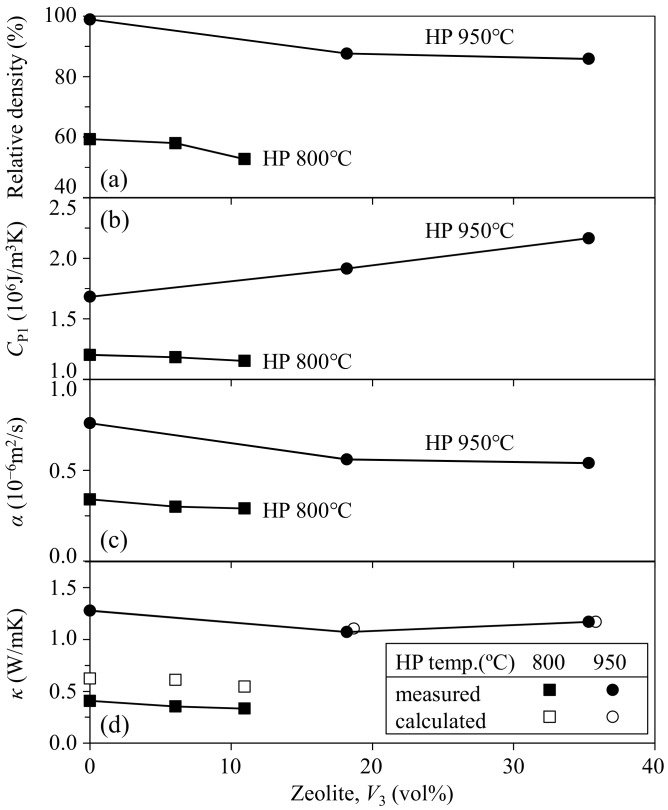
(**a**) Relative density; (**b**) heat capacity; (**c**) thermal diffusibility; and (**d**) thermal conductivity of zeolite–pore–shirasu glass composites at room temperature. The calculation of *κ* values for the composites was carried out using the thermal conductivity of 2.07 W/mK for mordenite zeolite.

Figure 5 shows the relative density, heat capacity, thermal diffusibility, and thermal conductivity for the mordenite–pore–shirasu glass composites hot-pressed at 800 °C or 950 °C. The relative density was in the ranges of 52.7–59.3% and 85.9–98.9% for the composites hot-pressed at 800 and 950 °C, respectively, and decreased gradually with increasing zeolite fraction. In the composite hot-pressed at 950 °C, two samples were fabricated for each zeolite content. The relative densities at the zeolite fractions of 0, 20.8, and 41.1 vol % were 98.81 ± 0.08%, 87.27 ± 0.31%, and 85.77 ± 0.07%, respectively, and were within the symbol size in Figure 5. The increased relative density at a higher hot-pressing temperature (950 °C) enhanced the heat capacity, thermal diffusibility, and thermal conductivity. As seen in Figure 5b, the mixing of the zeolite was effective to increase the heat capacity of the composite hot-pressed at 950 °C. However, the thermal diffusibility of the composite decreased at a higher fraction of the zeolite. The thermal conductivity (product of *C*_P1_ and *α*) was in the ranges of 0.334–0.409 W/mK and 1.07–1.28 W/mK for the composites hot-pressed at 800 and 950 °C, respectively, and almost independent of zeolite fraction. The *κ* values for 100% dense shirasu glass and zeolite particles were calculated by Equations (1) and (2), respectively, using the measured *κ* value for shirasu glass with 1.1% porosity (1.28 W/mK, hot-pressed at 950 °C) and the composite with 35.3 vol % zeolite–14.2 vol % porosity (1.17 W/mK, hot-pressed at 950 °C). The C element detected in Figure 3 and Figure 4 was treated as one component included in the shirasu glass continuous phase. The calculated values were 1.34 W/mK for 100% shirasu glass and 2.07 W/mK for the mordenite zeolite. Similarly, the *κ* value of the mordenite zeolite for 18.2 vol % zeolite–12.4 vol % pore–shirasu glass composite (1.07 W/mK) was calculated to be 1.70 W/mK. That is, the calculated *κ* values (1.70–2.07 W/mK) for the mordenite zeolite in the zeolite–pore–shirasu glass composites were relatively low as compared with the *κ* value (3.63 W/mK) of the mordenite in the zeolite–pore–phenol resin composite. One possible reason is the partial degradation of the mordenite structure due to the high temperature processing. Therefore, the degradation of the zeolite structure may be discussed from the point of view of the decrease in the thermal conductivity of zeolite.

**Figure 6:**
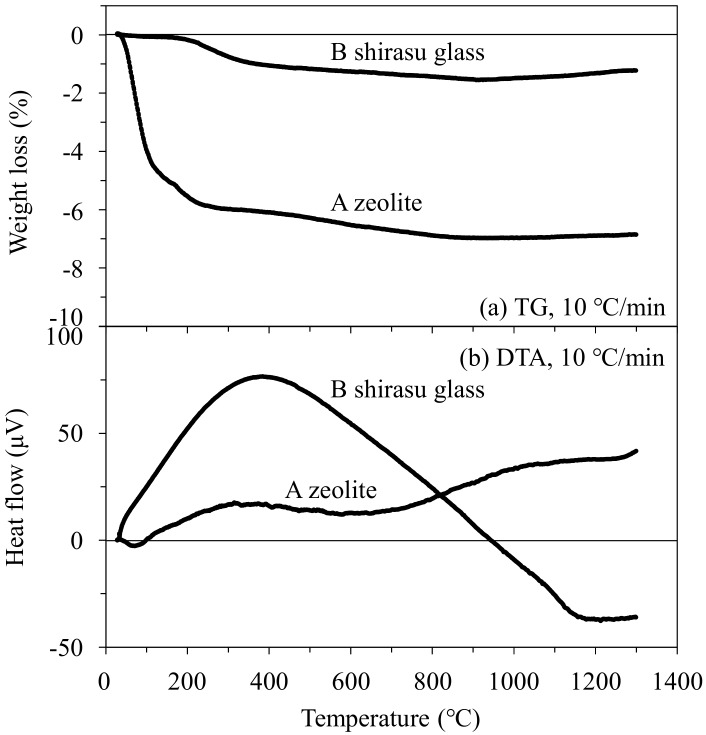
Thermogravimetric/differential thermal analysis (TG/DTA) curves for (A) mordenite zeolite and (B) shirasu glass.

Figure 6 shows the results of thermogravimetric/differential thermal analysis (TG/DTA) of mordenite zeolite and shirasu glass powders. A large weight loss of zeolite powder was measured up to 200 °C and then a small weight loss continued to 1000 °C. In the shirasu glass powder, a small weight loss of about 1% was measured at 200–300 °C. As seen in the DTA data, no significant endothermic and exothermic peaks associated with the crystallization of glass or decomposition of zeolite were observed up to 1300 °C. The degradation of the mordenite structure at high temperatures was not detected by the present DTA data.

**Figure 7:**
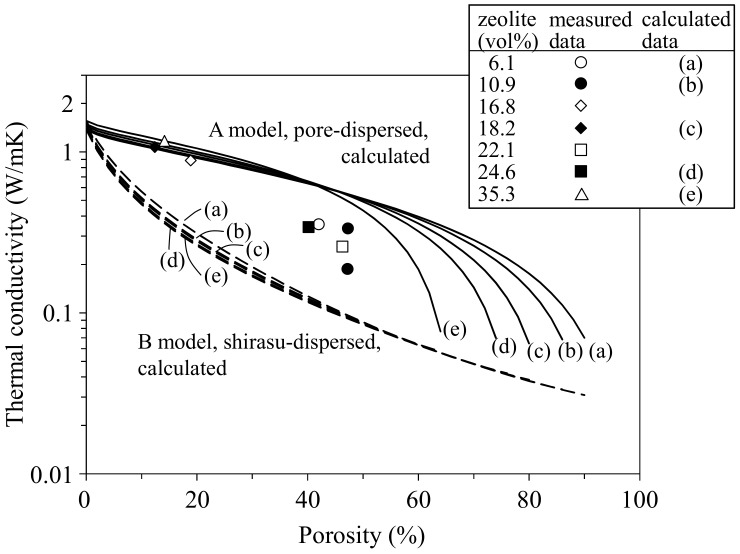
Relationship between the thermal conductivity and porosity for the mordenite zeolite–pore–shirasu glass composites.

Figure 5d shows the comparison of *κ* values in the experiment and calculation for the composites hot-pressed at 800 °C. Equation (2) with *κ* values of 1.34 W/mK for shirasu glass, 0.0265 W/mK for air, and 2.07 W/mK for mordenite zeolite was used for different volume fractions of the mordenite and air. As seen in Figure 5d, the calculated *κ* values exhibited a similar tendency with the volume fraction of the mordenite but were slightly higher than the measured *κ* values. This difference in the *κ* values is discussed with the result in Figure 7.

Figure 7 shows the relationship between the thermal conductivity and porosity for the zeolite–pore–shirasu glass composites at room temperature. The *κ* values calculated by Equation (2) for the zeolite–pore–shirasu glass three phase system are also plotted in Figure 7. In model structure A with a shirasu glass continuous phase, the calculated *κ* values decrease gradually with increasing porosity, and then drop rapidly around the critical porosity where the shirasu glass content approaches 0 vol %. In contrast, model structure B with a pore continuous phase shows the rapid decrease in *κ* values at a low porosity. The measured *κ* values at about 15 vol % porosity (0% closed pores and 12.4–14.2% open pores) were in good agreement with the calculated *κ* values for model structure A with the same porosity (closed pores). In this porosity range from 0 to 15%, the open pores convey the heat energy like closed pores. At a porosity higher than 40%, the measured *κ* values were observed below the calculated *κ* values for model structure A and approached the *κ* values for model structure B. The composites with a total porosity of 40.2–47.3 vol % contained 29.3–46.8% open pores and 0.4–12.7% closed pores. The increased fraction of open pores causes the deviation from the calculated *κ* value curves for model A. In the hot-pressed porous composites, model structure B with continuous open pores was formed partially. A similar porosity dependence of thermal conductivity was also observed in monolithic mullite compacts in our previous study [7]. This type of structure change with increasing porosity appears to be the essential feature of porous ceramics.

### 3.4. Temperature Dependence of Thermal Properties of the Zeolite–Pore–Shirasu Glass Composites

**Figure 8:**
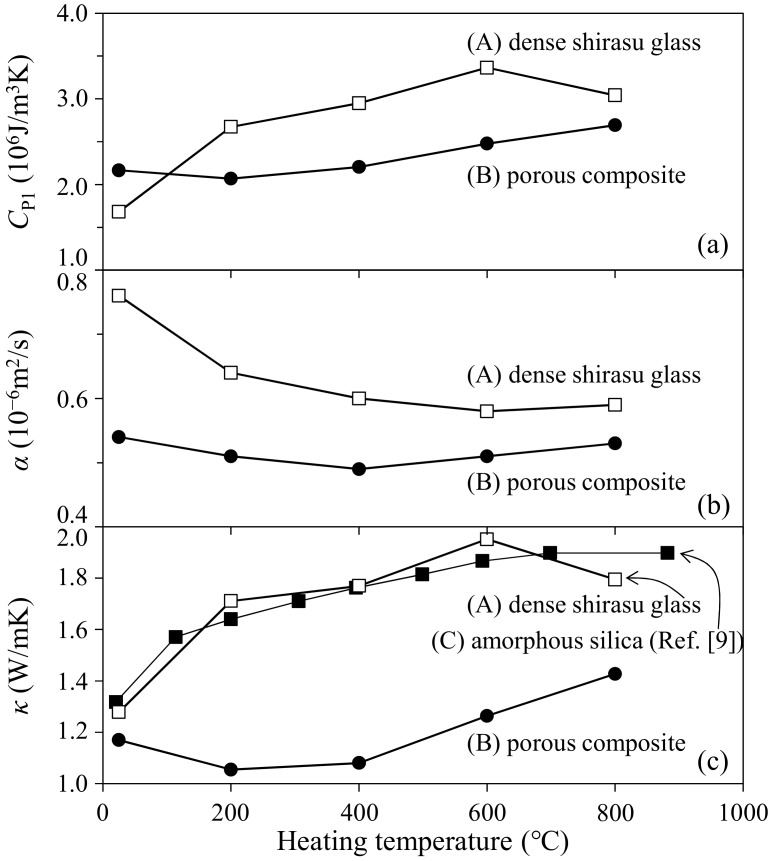
(**a**) Heat capacity; (**b**) thermal diffusibility; and (**c**) thermal conductivity of the monolithic shirasu glass with 1.1% porosity and the 35.3 vol % zeolite–14.2 vol % pore–shirasu glass composite.

Figure 8 shows the temperature dependence of heat capacity, thermal diffusibility, and thermal conductivity of the monolithic shirasu glass with 1.1% porosity and the 41.1 vol % zeolite–shirasu glass composite with 14.2% porosity. While the *C*_P1_ values of shirasu glass increased with increasing temperature, the *α* values decreased at a higher temperature. The variation of the *C*_P1_ values dominated the temperature dependence of the *κ* values of shirasu glass, as seen in Figure 8c. The magnitude and temperature dependence of the *κ* values of shirasu glass showed good agreement with the reported *κ* values (1.3–1.9 W/mK at 20–900 °C) of amorphous silica [9], supporting the validity of the present thermal conductivities measured in this paper. On the other hand, the porous zeolite–shirasu glass composite exhibited a smaller temperature dependence of the *C*_P1_, *α,* and *κ* values. This result is discussed as follows.

**Figure 9:**
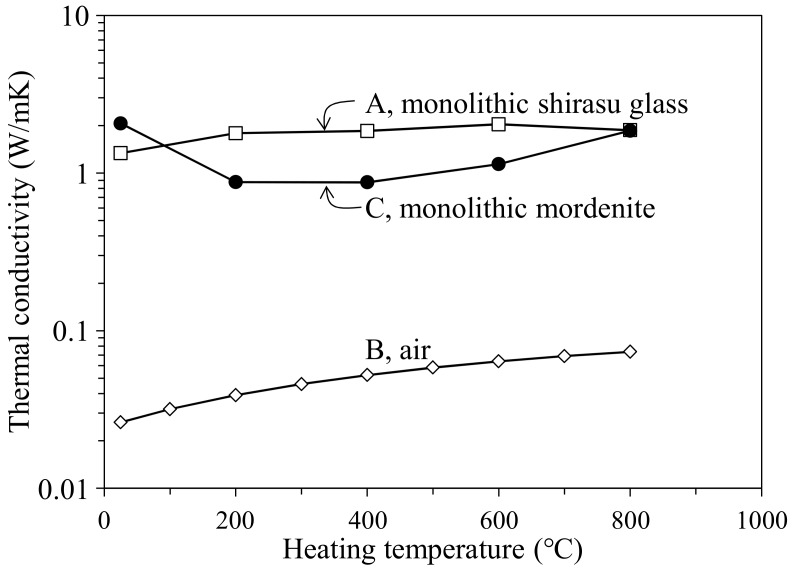
Dependence of the thermal conductivities of zeolite powder, shirasu glass, and air on heating temperature.

Figure 9 shows the temperature dependence of the thermal conductivities of monolithic shirasu glass without pores and air reported [8]. The data for shirasu glass were determined from Equation (1) for the measured *κ* values of the hot-pressed shirasu glass and air. The *κ* values of monolithic mordenite zeolite in the 35.3 vol % zeolite–14.2 vol % pores–shirasu glass composites were calculated by Equation (2) for model structure A (shirasu glass continuous phase) using the *κ* values for monolithic shirasu glass and air plotted in Figure 9. As seen in Figure 9, the *κ* value for mordenite is larger than that for shirasu glass at room temperature but decreases below 1 W/mK at 200–600 °C. These characteristics of the thermal properties of mordenite zeolite and the low *κ* values of air are combined to give the small temperature dependence of *κ* values for the zeolite–pore–shirasu glass composite, shown in Figure 8c. The open structure of zeolite may be responsible for the low *κ* values plotted in Figure 9.

The temperature dependence of the thermal conductivity of mordenite zeolite was clarified in the wide temperature range of 25–800 °C for the first time. As seen in Figure 6, the mordenite lost its significantly large weight by 200 °C and showed a further small weight loss up to 1000 °C. This weight loss is caused by the release of H_2_O, N_2_, and O_2_ molecules captured in the cage structure of mordenite zeolite. The *κ* value of the open spaces without captured molecules becomes lower because of the formation of vacuum spaces, resulting in the decrease of *κ* values of the monolithic mordenite (Figure 9) and the composite (Figure 8) with heating. This characteristic of the *κ* value of zeolite is interesting in applications as refractory materials.

## 4. Conclusions

The zeolite (mordenite)–pore–phenol resin composite and the zeolite–pore–shirasu glass composite were fabricated by hot-pressing at 150 °C and 800–950 °C, respectively. The measured thermal conductivities were analyzed with the proposed mixing rule of thermal conductivities of the component materials and air.

(1) The thermal conductivity of mordenite was calculated to be 3.63 W/mK at room temperature based on the thermal conductivities of a dense monolithic phenol resin and the dense 12.4 vol % zeolite–3.1 vol % pore–phenol resin composite. The relative density of the composite decreased at higher zeolite contents (>15 vol %). The thermal conductivities of the composites with 0–21.7 vol % zeolite and 0.7–23.0 vol % pores were almost constant and were in the range of 0.23–0.30 W/mK.
(2) In the zeolite–pore–shirasu glass composite, no change in the crystalline phases was observed after hot-pressing at 800–950 °C. The relative density of the composite decreased with increasing zeolite content and at a lower hot-pressing temperature. The thermal conductivities at room temperature were almost constant (1.07–1.28 W/mK) in the range from 0 to 35.3 vol % of zeolite content. This result is explained by the compensation effect between the zeolite with a higher thermal conductivity and air with a lower thermal conductivity.
(3) The thermal conductivity of the zeolite–pore–shirasu glass composite with less than 15 vol % porosity was in good agreement with the calculated thermal conductivity for the shirasu glass continuous structure (structure A). However, the thermal conductivity of the composite decreased from the calculated value for structure A in the porosity range higher than 40 vol % and approached the value calculated for structure B with a pore continuous phase structure.
(4) The thermal conductivity of the monolithic mordenite was calculated for the composite hot-pressed at 950 °C to be 1.70–2.07 W/mK, and these values were smaller than the thermal conductivity (3.63 W/mK) of the zeolite processed at 150 °C. The decreased thermal conductivity of mordenite processed at high temperatures may reflect the partial decomposition of zeolite.
(5) The thermal conductivity of the 35.3 vol % zeolite–14.2 vol % pore–shirasu glass composite exhibited the minimum value of 1.06 W/mK at 200 °C and then increased to 1.43 W/mK at 800 °C. The above result is strongly related to the change of the thermal conductivity of monolithic mordenite with heating. With heating, H_2_O, O_2_, and N_2_ molecules captured in the cage structure of mordenite are released, leading to the decrease in the thermal conductivity due to the formation of vacuum spaces in the mordenite.

## References

[1] Coquil T., Lew C.M., Yan Y., Pilon L. (2010). Thermal conductivity of pure silica MEL and MFI zeolite thin films. J. Appl. Phys..

[2] McGaughey A.J.H., Kaviany M. (2004). Thermal conductivity decomposition and analysis using molecular dynamics simulations Part II. complex silica structures. Int. J. Heat Mass Trans..

[3] Griesinger A., Spindler K., Hahne E. (1999). Measurements and theoretical modelling of the effective thermal conductivity of zeolites. Int. J. Heat Mass Trans..

[4] Hirata Y. (2009). Representation of thermal conductivity of solid material with particulate inclusion. Ceram. Int..

[5] Hirata Y., Fukushige Y., Kuwazuru H., Yamashita R., Sameshima S., Kamino Y. (1997). Electrical properties of carbon fiber/shirasu glass composite. J. Phys. Chem. Solids.

[6] Itoh S., Hirata Y., Shimonosono T., Sameshima S. (2015). Theoretical and experimental analyses of thermal conductivity of the alumina–mullite system. J. Eur. Ceram. Soc..

[7] Hirata Y., Kinoshita Y., Shimonosono T., Chaen T. (2017). Theoretical and experimental analyses of thermal properties of porous polycrystalline mullite. Ceram. Int..

[8] Hata K. (1984). Chemical Handbook.

[9] Freeman J.J., Anderson A.C. (1986). Thermal conductivity of amorphous solids. Phys. Rev. B.

